# Automatization and stress analysis data of CoCr laser weld fatigue tests

**DOI:** 10.1016/j.dib.2019.104374

**Published:** 2019-08-27

**Authors:** M. Kanerva, Z. Besharat, T. Pärnänen, J. Jokinen, M. Honkanen, E. Sarlin, M. Göthelid, D. Schlenzka

**Affiliations:** a Tampere University, Faculty of Engineering and Natural Sciences, P.O. Box 589, FI-33014 Tampere, Finland; b Orton Orthopaedic Hospital and Research Institute Orton, FI-00280 Helsinki, Finland; c Royal Institute of Technology, Surface and Corrosion Science, P.O. Box 10044, SE-16440 Stockholm, Sweden

**Keywords:** Fatigue, Automatization, Testing, CoCr, Welding, Laser

## Abstract

This work includes raw and analyzed test data when using a recently developed fatigue test method for miniature laser welds in cobalt-chromium (CoCr) alloy joints [1]: 10.1016/j.jmbbm.2019.07.004. The automization of fatigue tests is crucial for saving costs and personnel resources and that is the reason why the atomization threshold and the resulting spectrum data related to CoCr welds are provided here. The finite element method based stress computation output is provided related to shearing-mode tests to support the dataset as a whole. In addition, the compositional data of the parent material and the laser weld are given.

**Table:** Specifications Table

Subject area	*Materials Science*
More specific subject area	*Metals and Alloys, Laser welds, Medical devices*
Type of data	*Graphs, Text files, Figures*
How data was acquired	*Mechanical testing: Electropuls E 3000, Instron; WaveMatrix, Instron* *Finite element computation: ABAQUS by Dassault Systemes, standard/2017* *Energy dispersive X-ray spectroscopy: X-Max SDD*
Data format	*Raw and analyzed, visualizations*
Experimental factors	*Industrial and clean room surface cleaning for CoCr*
Experimental features	*Laser welds at the length scale of 100 μm*
Data source location	*Espoo, Finland (Synoste Oy); Tampere, Finland (Tampere University); Stockholm, Sweden (Royal Institute of Technology)*
Data accessibility	*Data is available from the corresponding author upon reasonable request*
Related research article	*M. Kanerva, Z. Besharat, T. Pärnänen, J. Jokinen, M. Honkanen, E. Sarlin, M. Göthelid, D. Schlenzka. Miniature CoCr laser welds under cyclic shear: Fatigue evolution and crack growth. Journal of the Mechanical Behavior of Biomedical Materials, 99, 2019, 93–103* https://doi.org/10.1016/j.jmbbm.2019.07*.004* [1]

**Table:** 

**Value of the Data** • The data can improve the efficiency of fatigue test programming of laser welds under shear load in universal test machines • The stress data can help to understand and improve the geometry and parameters of miniature laser welds at the length scale of 100 μm • The compositional results provide information about the CoCr alloy to improve surface quality due to laser welding in medical devices

## 1 Data

Data of the atomization is describing the stiffness (deformation) changes in the specimen and the machine stop at different phases of testing. The data of all the graphs below are also given as source data files. The finite element method based stress computation output is given as filtered, visualized fields and as a complete output database. The composition data are given as comparative plots and source data files.

## 2 Experimental design, materials, and methods

### 2.1 Data of the fatigue test automatization

**Fig. 1:**
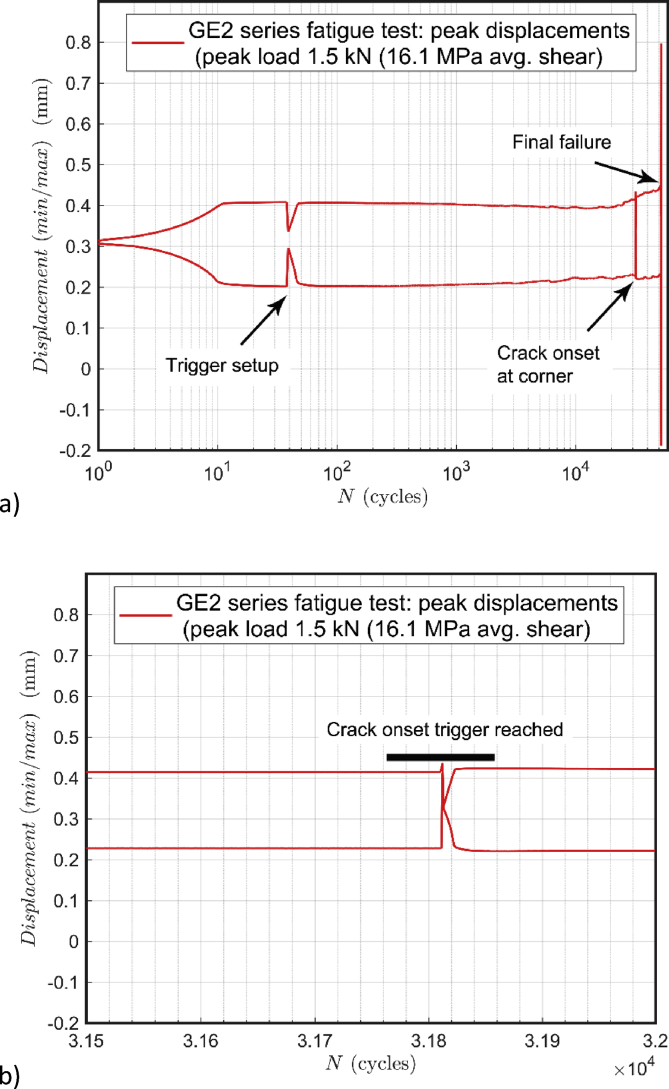
The progress of displacement (weld strain hardening and damage) during a fatigue test: a) entire fatigue test; b) portion of automatic triggering at crack onset (till the weld corner). The complete number-form input data of the graphs is included (mmc4.xlsx).

The fatigue testing was performed according to the test procedure and specimen preparation as described in a previous work [1]. In detail, the test specimen geometry GE2 (i.e. the G2 design in Ref. [1]) was used in the reported test data here. The testing machine (Electropuls E3000, Instron) was used with a 3 kN load cell and integrated computerized control (WaveMatrix, Instron). The GE2 fatigue test specimens were tested in two phases including: (1) damage onset phase, and (2) shear fatigue phase. Automatization of the phase-division was set by using a digitized trigger according to the drift in the maximum displacement during the test. The damage onset at one end of the specimen (welding point 7 and/or 8 as given in Ref. [1]) results in a crack and that leads to the test machine-recorded displacement to grow; a 5% threshold was used to identify crack propagation until the corner where the main shear-loading part begins. Each test and threshold triggering was verified visually by using a microscope. Typical cycle minima-maxima curves are given in Fig. 1. As a phase definition in data, the final failure, determining the fatigue life of the specimen, is established by a sudden and intensive growth of the displacement (level).

### 2.2 Data of the weld's numerical stress computation

**Fig. 2:**
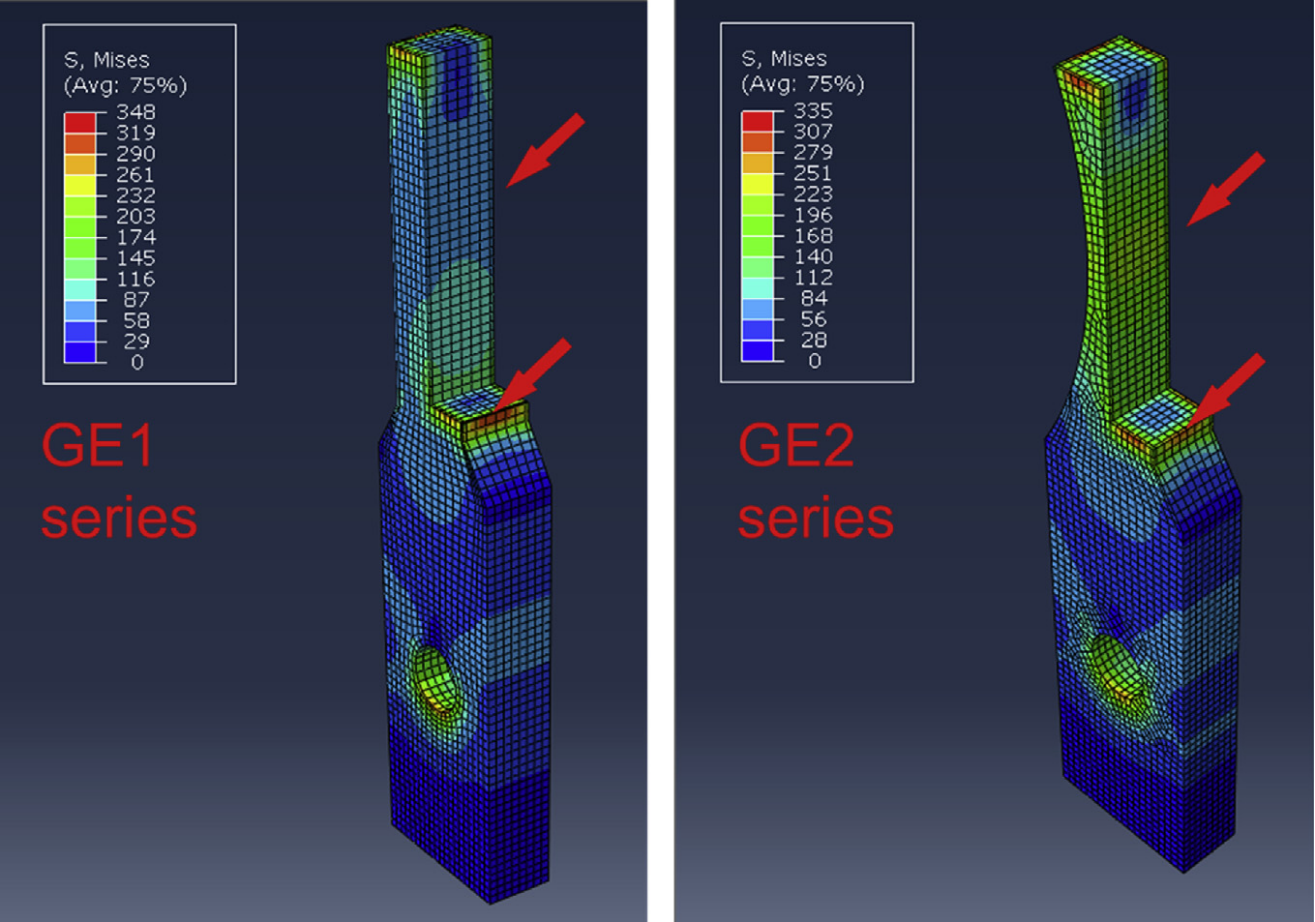
Stress computation data and visualization between the specimens geometries GE1 and GE2 when the entire weld is intact. The applied load in the visualized computation is 2 kN. The complete output files are accompanied in this work (mmc2: FEM_ij.odb; i = geometry (GE1/2), j = crack/nocrack).

**Fig. 3:**
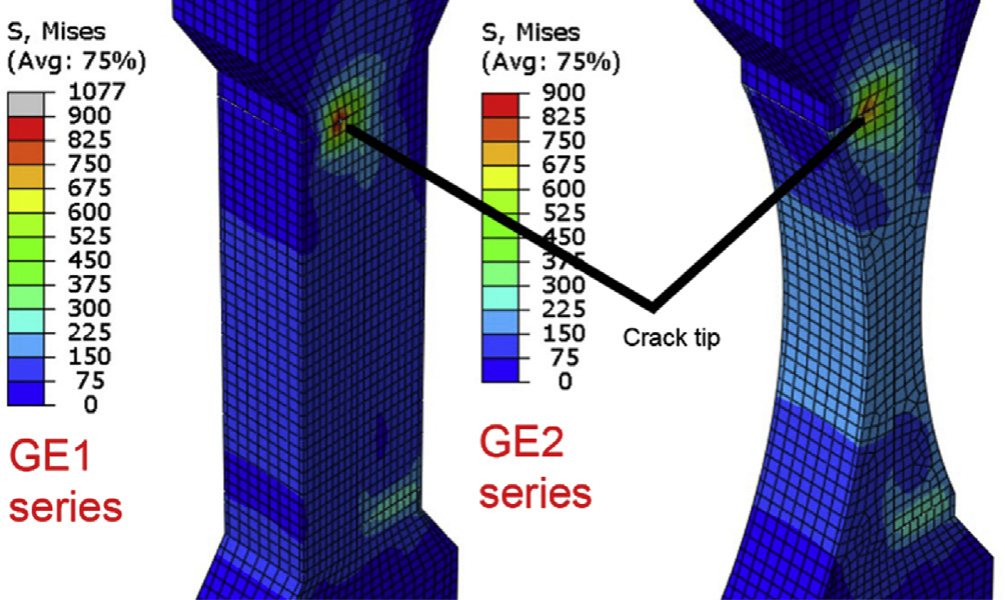
Stress computation data and visualization between the specimens GE1 and GE2 when the upper corner-weld has crack towards the corner (1.5 mm crack along the side). The applied load in the computation is 2 kN. The complete output files are accompanied in this work (FEM_ij.odb; i = geometry, j = crack).

The numerical stress computation was conducted using a commercial ABAQUS standard/2017 [2] software code. The material models were configured as described in Ref. [1] so that the GE2 (G2 [1]) and GE1 (i.e. G1 in Ref. [1]) specimen geometries were created using the original CAD files given as Appendix in Ref. [1]. The computations were run for intact specimens (crack length 0 mm) and for a manual crack opening at one end (1.5 mm). Standard stress computations were run with a 2 kN load subjected at the specimen boundary and von Mises stresses were filtered from the total output data to produce the fields illustrated in Fig. 2, Fig. 3.

### 2.3 Compositional data of laser welds

**Fig. 4:**
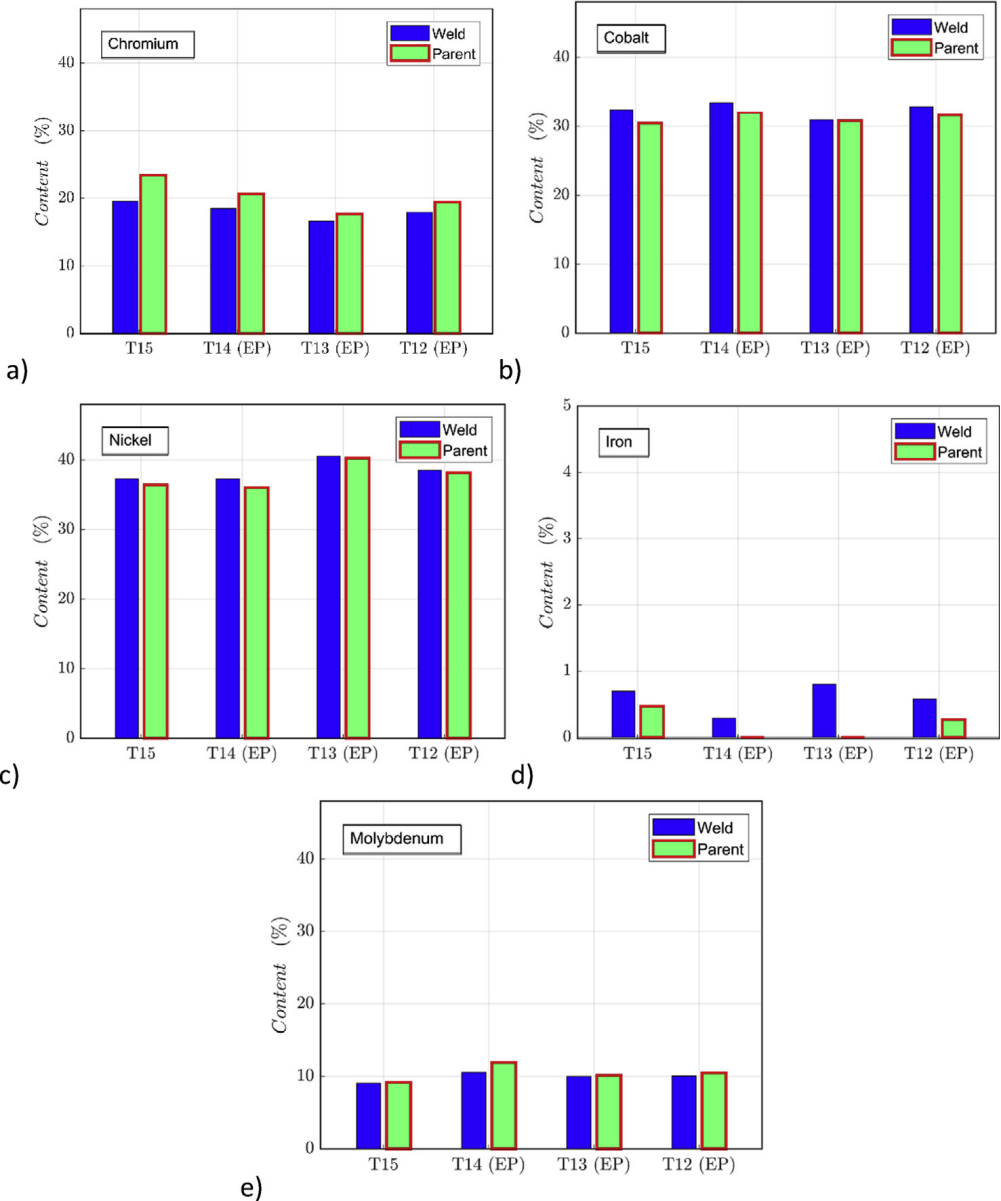
EDS compositional data measured for weld seam and parent material in four test specimens with or without EP treatment: a) chromium content; b) cobalt content; c) nickel content; d) iron content; e) molybdenum content. The complete data of the graphs is included (mmc1, mmc3: CoCrcomposition.m, with input files COLMi.txt where i = graph number).

Energy dispersive X-ray spectroscopy (EDS) was run on the surfaces of parent (GE2) specimen halves and on the laser weld by using EDS X-Max SDD (Silicon Drift Detector). Prior to welding, the specimens went through a nine-step cleaning process as described in Ref. [1]. The EDS data for four different specimens (entitled T12, T13, T14, T15) are given Fig. 4. Part of the measured specimens were electropolished (EP) for further surface characterization (see specimens with 'EP' in Fig. 4).
